# Erianin: A phytoestrogen with therapeutic potential

**DOI:** 10.3389/fphar.2023.1197056

**Published:** 2023-08-07

**Authors:** Gangmin Li, Huiqiong Zhang, Hui Lai, Gang Liang, Jiang Huang, Fulan Zhao, Xiaofang Xie, Cheng Peng

**Affiliations:** ^1^ Department of Pharmacy, The Affiliated Traditional Chinese Medicine Hospital of Southwest Medical University, Luzhou, China; ^2^ Safety Evaluation Center, Sichuan Institute for Drug Control (Sichuan Testing Center of Medical Devices), Chengdu, China; ^3^ State Key Laboratory of Traditional Chinese Medicine Resources in Southwest China, Chengdu University of Traditional Chinese Medicine, Chengdu, China

**Keywords:** erianin, anti-tumour effect, mechanism, pharmacokinetics, derivatives

## Abstract

Erianin, a phytoestrogen with therapeutic potential, is one of the major active components of Dendrobll caulis. Erianin has a variety of pharmacological effects, such as anti-tumor, anti-inflammatory, anti-diabetic retinopathy, anti-psoriasis, and antibacterial effects. Especially, in regard to the anti-tumor effect of erianin, the underlying molecular mechanism has been partly clarified. In fact, the numerous pharmacological actions of erianin are complex and interrelated, mainly including ERK1/2, PI3K/Akt, JAK2/STAT3, HIF-1α/PD-L1, PPT1/mTOR, JNK/c-Jun, and p38 MAPK signal pathway. However, on account of the poor water solubility and the low bioavailability of erianin, greatly affected and limited its further development and application. And it is worthwhile and meaningful to explore more extensive pharmacological effects and mechanisms, clarify pharmacokinetics, and synthesize the derivatives of erianin. Conclusively, in this paper, the pharmacological effects of erianin and its mechanism, pharmacokinetics, and derivatives studies were reviewed, in order to provide a reference for the development and application of erianin.

## 1 Introduction

**FIGURE 1:**
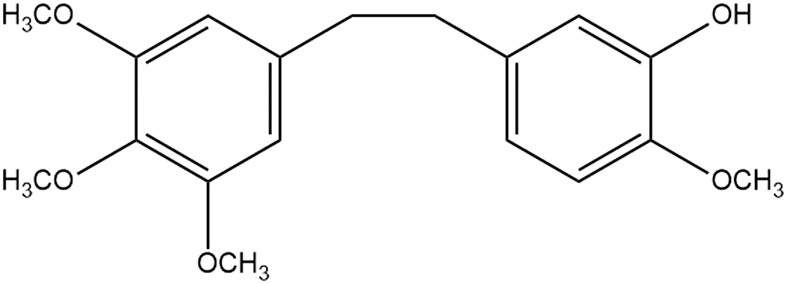
Chemical structure of erianin

Traditional Chinese medicine Dendrobll caulis is mainly derived from Dendrobium SW. of Orchidaceae, and is cultivated as a fresh or dried stem of the *Dendrobium nobile* Lindl., *Dendrobium huoshanense* C. Z. Tang et S. J. Cheng, *Dendrobium chrysotoxum* Lindl., or *Dendrobium fimbriatum* Hook., respectively (National Pharmacopoeia Commission, 2020). Erianin (C_18_H_22_O_5_), chemically named 2-methoxy-5-[2-(3,4,5-trimethoxy-phenyl)-ethyl]-phenol, belonging to the benzidine compound. And the structural formula is shown in Figure 1. What is noteworthiness is that erianin is a small molecule compound isolated from the TCM Dendrobll caulis and the main active ingredient of its pharmacological effects (ZHANG Y. et al., 2019; Ming et al., 2022). Currently, A large number of studies have demonstrated that erianin had extensive and potent pharmacological activities, such as anti-tumor, anti-inflammatory, anti-diabetic retinopathy, antibacterial, and antipsoriatic effects, among others (Zhang et al., 2018; Ming et al., 2022), and the pharmacological effects and related mechanisms are summarized in Table 1. In fact, erianin has the strongly effective antitumor effect, which is mainly mediated by promoting apoptosis, inducing cell cycle arrest, inducing cell autophagy, promoting ferroptosis, and inhibiting angiogenesis (Li S et al., 2019). Besides, erianin had the characteristics of low bioavailability and poor water solubility, hence clarifying its potential dose-effect relationship, and improving its structure to increase its bioavailability, which are of great significance for its utilization. Especially, the current pharmacological effects of erianin and its mechanism have not been fully elucidated. Therefore, the pharmacological properties, potential mechanisms, pharmacokinetics of erianin as well as effects of erianin derivatives will be reviewed and analyzed in detail in this review.

**TABLE 1:** Pharmacological mechanism of erianin

Effects	Pathways	Dosage	Diseases/Cells	Mechanism	References
Anti-tumor effect	Apoptosis				
	ERK1/2	3.9, 7.8, 15.7, and 31.4 μM	Cervical carcinoma	p53, caspase 3, and Bax **↑**, Bcl-2 and ERK1/2 phosphorylation **↓**	LI et al. (2018)
			HeLa cells		
		20, 40, and 80 nM	Nasopharyngeal carcinoma	death receptors (Fas, TNF-R2, DcR2, DR5 and RIP) and cleaved caspase-3, -8, and -9 **↑**, ERK1/2 phosphorylation **↓**	LIU et al. (2019)
			NPC-039 and NPC-BM cells		
	Nrf2/NF-κB	40 and 80 nM	Liver cancer	*In vitro*, apoptosis and ROS release **↑**, mitochondrial membrane potential **↓**, and caspase-3, - 8, - 9, and PARP **↑**	ZHANG et al. (2019b)
		20 mg/kg	HepG2 and SMMC-7721 cells	*In vivo*, the level of MMP-2, -9, and IL-10 **↓**, Nrf2, HO-1, SOD-1, and SOD-2 **↑**, the phosphorylation of ERK1/2, IKK α/β and NF-κB **↓**	
			Tumor-bearing nude mice		
	JNK-Mitochondrial apoptosis	40, 60, and 80 nM	Bladder cancer	*In vitro*, Bim, Bad, p-JNK, p-Bcl-2, p-c-Jun ↑, Bcl-2 and Mcl-1↓	ZHU et al. (2019)
		25, 50, and 100 nM	EJ cell/T24 cell	*In vivo*, suppressing tumor growth and increasing eosinophils expression	
		50 mg/kg	Tumor-bearing nude mice		
	PI3K/Akt	40, 80, and 160 nM	Breast cancer	Cyt-c, PARP, Bax, cleaved caspase-3, and -9 ↑, p-PI3K and p-Akt ↓	XU et al. (2021)
			MDA-MB-231 and EFM-192A cells		
		19.6, 37.3 and 78.5 nM	Liver cancer	the cleaved PARP ↑, p-Akt and Mcl-1 ↓	Su et al. (2011)
			Huh7 cell		
		10 and 20 μM	Lung cancer	p-Akt, p-ERK, and p-p38 ↓	YANG et al. (2020)
			HCC cell		
	JAK2/STAT3	50, 100, and 200 nM	Colon cancer	Bcl-2, CyclinD1, C-Myc, p-STAT3, and P-gp ↓	SU et al. (2021)
			HCT116/LOHP cell		
	HIF-1α/PD-L1	10, 30 and 100 μM	Cervical carcinoma	promoting the degradation of lysosomes in Hela cells, further inhibiting the expression of PD-L1, the expression of p-c-Raf、p-MEK、p-ERK、p-p38 and p-JUK ↓	YANG et al. (2021)
			Hela cell		
	PPT1/mTOR	50 and 100 nM	Oral Cavity Cancer	*in vivo* and *in vitro*, PPT1, p-mTOR, and p-4EBP1 ↓	LUO et al. (2021)
		25 and 100 mg/kg	WSU-HN4, SCC-9 and CAL-27 cells		
			Tumor-bearing nude mice		
	JNK/c-Jun	20, 40, and 60 nM	Breast cancer	PARP, caspase-3, caspase-8 and bax ↑. However, these effects could be partially reversed by JNK inhibitor SP600125	Zhao et al. (2020)
			MDA-MB-231 and MDA-MB-468 cells		
	p38 MAPK	5, 10, and 20 μM	Melanoma	p38 MAPK, and PARP ↓, p21, cleaved PARP, and cleaved caspase-3 ↑	Guiping and Wang (2019)
			C918 and MUM-2B uveal melanoma cells		
		20, 40, and 80 nM	Leukemia	p-p38, γ H2AX, cleaved caspase-3, -9, and Bax ↑	Xuyan et al. (2020)
			Jurkat cell		
	Cell cycle	10, 25, and 50 nM	Osteosarcoma	cyclin B1, p-CDK1, p-Cdc25c, p21 and p27 ↑	WANG et al. (2016)
			143B and MG63.2 cells		
		12.5, 25, 50, and 100 nM	Prostatic cancer	cyclin B1 and CDK1 ↑	TRAPIKA et al. (2021)
			LNCaP cell		
	Autophagy	25, 50, and 100 μM	Oral Cavity Cancer	LC3-I and LC3-II ↑, p62 ↓	CHEN et al. (2020a)
			SAS and SCC9 cells		
	Ferroptosis	12.5, 25, 50, and 100 nM	Lung cancer	HO-1, GPX4, CHAC2, SLC40A1, SLC7A11, glutaminase ↓, Transferrin ↑. But ferroptosis inhibitors Fer-1 and Lip-1 could reverse Erianin induced death	CHEN et al. (2020b)
			H460 and H1299 cells		
	Angiogenesis	10 mg/kg	Tumor-bearing mice chicken embryos	inhibiting angiogenesis and tumor weight in tumor bearing mice, disrupting endothelial tube formation, and abolishing collagen migration and adhesion to fibronectin	GONG et al. (2004a)
		10 nM			
		10 nM	HUVECs	lactic acid production, glucose consumption and intracellular ATP content ↓, but above situation were significantly reversed by JNK/SAPK inhibitor SP600125	GONG et al. (2004b)
		45, 90, and 180 nM	HUVECs and 2LL cell	blocked tube formation in HUVECs	SU et al. (2017)
				p-STAT3, p-JAK2, MMP-2, MMP-9, COX-2, HIF-1α, and IL-6 in 2LL cells ↓	
Anti-inflammatory effect	TLR4/NF-κB/STAT3	10 and 20 mg/kg	UC mice	ROS and ROS in the serum↓, TLR4, TRAF6, p-IKK α/β, p-IκB α, NF-κB p65, p-JAK2, p-Akt, and p-STAT3↓	DOU et al. (2020)
	NLRP3	1, 5, and 25 nM	WT mice	inhibiting the activation of NLRP3 inflammasome *in vitro* and *in vivo*, and blocking the assembly of NLRP3 inflammasome	ZHANG et al. (2021b)
Anti-diabetic retinopathy effect	ERK1/2–NF-κB	1 and 10 mg/kg	STZ-induced diabetic mice	p-IκB, p-IKK, p-p65, and TNF-α and p-ERK1/2/t-ERK1/2 ratio *in vitro* and *in vivo* ↓	ZHANG et al. (2019c)
	ERK1/2-HIF-1α-VEGF/VEGFR2	5, 10, 25, and 50 Nm	BV-2 cells	VEGF, HIF-1 α., p-ERK1/2, p-VEGFR2, p-c-Raf, p-MEK1/2, p-p38, p-Akt, p-PI3K, p-mTOR, and p-P70S6kinase↓	YU et al. (2016)
		1 and 10 mg/kg	STZ-induced hyperglycemic mice		
Antibacterial effect		8, 16, 32, and 64 μg/mL	taphylococcus aureus	inhibited the activity of *staphylococcus aureus* binding to fibronectin and biofilm formation	OUYANG et al. (2018)
Antipsoriasis	JNK/c-Jun/Akt/mTOR	12.5, 50, and 100 nM	HaCaT cells	cleaved PARP, cleaved caspase-3, p-JNK, p-c-jun and decreased p-Akt, p-mTOR↑, while these effects were also reversed with NAC	MO et al. (2019)

## 2 Pharmacological activities

### 2.1 Anti-tumor effect

#### 2.1.1 Cell apoptosis

**FIGURE 2:**
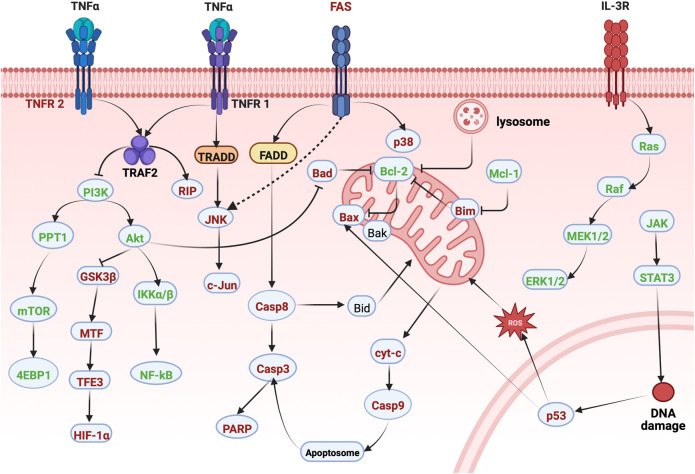
Molecular pathways involved in the apoptosis of erianin.

For decades, many scholars and experts have bent their efforts toward the effective elimination of tumor cells by promoting apoptosis, which is convenient for going a step further to achieve the effect of cancer treatment in clinical practice (D'ARCY, 2019). The pathway of apoptosis mainly includes cell intrinsic and extrinsic apoptotic processes resulting from DNA damage, destruction of cell structure, and dysregulation of regulatory proteins function (PISTRITTO et al., 2016; Obeng, 2021). Regulation of pro-apoptosis factors (Bax, etc.) and anti-apoptosis factors (Bcl-2, Bcl-xL, etc.) activate or inhibit their up - and downstream signaling pathways to exert anti-tumor effects through their promotion of apoptosis (GOLDAR et al., 2015). Notwithstanding, part of the potential signaling pathways have been studied in detail, including ERK1/2, Nrf2/NF-κB, PI3K/Akt, JAK2/STAT3, HIF-1α/PD-L1, PPT1/mTOR, JNK/c-Jun, p38 MAPK, and JNK-Mitochondrial apoptosis signal pathway. The apoptosis molecular targets of erianin mentioned in this article are shown in Figure 2.

##### 2.1.1.1 ERK1/2

ERK1 and ERK2, crucial protein kinases of the RAS/Raf/MEK/ERK/MAP signaling pathway, participated in and regulated various cellular processes in unison, including cell proliferation, apoptosis, immune response, synthesis and processing of RNA, and so on (ROSKOSKI, 2019). However, the deregulation of ERK1/2’s activity may be the hallmark of cancer. Sustained and marked activation ERK1/2 pathway could enhance apoptosis to induce tumor cell death (Sugiura et al., 2021). Noteworthily, it was reported that erianin (3.9, 7.8, 15.7, and 31.4 μM) inhibited HeLa cell survival in a dose- and time-dependent manner, including apoptosis and G_2_/M arrest. In particular, at doses of 15.7 and 31.4 μM, erianin significantly upregulated p53, caspase 3, and Bax, while downregulated Bcl-2 and ERK1/2 phosphorylation (LI et al., 2018). Additionally, compared with non-treated cells, erianin at 20, 40, and 80 nM could inhibit the survival of nasopharyngeal carcinoma cells (NPC-039 and NPC-BM), apoptosis, and G_1_ phase arrest in a dose- and time-dependent manner. 80 μmol/L erianin observably activated death receptors (Fas, TNF-R2, DcR2, DR5 and RIP), cleaved caspase-3,-8,-9, while notably reduced ERK1/2 phosphorylation (LIU et al., 2019). In general, these studies indicated that ERK1/2 takes part in the regulation of several pathways (p53 pathway and death receptor pathway, etc.), and inhibition of ERK1/2 is clearly beneficial for the promotion of apoptosis and tumor therapy.

##### 2.1.1.2 Nrf2/NF-κB

Oxidative stress induces excessive release of ROS and dysfunction of mitochondrial metabolism, which in turn leads to mitochondrial apoptosis (BOLISETTY et al., 2013). Certainly, nuclear factor erythroid 2-related factor 2 (Nrf2), a pivotal factor in oxidative stress, promoted HO-1 expression and impaired mitochondrial membrane potential (BANSAL et al., 2014). In parallel, oxidative stress activates nuclear factor κB (NF-κB), which aggravates inflammation via preventing matrix metalloproteinases (MMPs) (Shi et al., 2015) and facilitating the expression of interleukins (LIANG et al., 2017). Simultaneously, the study showed that erianin could reduce the activity of HepG2 and SMMC-7721 cells, inhibit their proliferation and migration, and induce G_2_/M phase arrest. 80 μmol/L erianin significantly enhanced apoptosis and ROS release, while observably decreasing mitochondrial membrane potential. And 80 μM erianin also upregulated the expression of caspase-3, - 8, - 9, and PARP. In addition, compared with the model group, 20 mg/kg of erianin could dramatically restrain the tumor growth of tumor-bearing mice, and significantly reduced the level of MMP-2, -9, and IL-10 in the serum. Moreover, erianin strongly increased Nrf2, HO-1, SOD-1, and SOD-2, while decreasing the phosphorylation of ERK1/2, IKK α/β, and NF-κB *in vivo* (ZHANG X. et al., 2019). Even more momentously, activating the Nrf-2/NF-κB pathway is also of great significance in relieving oxidative stress and inflammation, which is helpful for cancer treatment by promoting apoptosis.

##### 2.1.1.3 JNK-mitochondrial apoptosis

The JNK pathway plays a crucial role in various physiological processes, especially apoptosis, cell proliferation, cell differentiation, and inflammation (KUMAR et al., 2015). Simultaneously, the JNK pathway also regulates the intrinsic pathway (mitochondria-mediated pathway) (SINHA et al., 2013), which mainly includes altering mitochondrial membrane potential, promoting the release of Cyt-c, and the expression of related proteins and disrupting mitochondrial metabolic function (YIN et al., 2013). Moreover, Zhu et al. reported that erianin significantly inhibited the proliferation of EJ and T24 cells, and induced G_2_/M phase arrest. And erianin could promote Cyt-c release and alter mitochondrial membrane potential. Besides, erianin upregulated the expression of Bim, Bad, p-JNK, p-Bcl-2, p-c-Jun, but downregulated the expression of Bcl-2 and Mcl-1. In addition, 50 mg/kg of erianin could obviously suppress tumor growth and increase eosinophils expression in tumor-bearing nude mice (ZHU et al., 2019).

##### 2.1.1.4 PI3K/Akt

PI3K/Akt signaling pathway is one of the major signaling pathways in apoptosis, which has been emphasized especially in intracellular proliferation, growth, and survival (ALZAHRANI, 2019). Multiple studies strongly demonstrate that the PI3K/Akt signaling pathway is commonly activated in various human cancer diseases. Surprisingly, some PI3K inhibitors are approved for the clinical treatment of human cancers, suggesting that the PI3K/Akt signaling pathway has an undeniable role and unlimited potential in tumor targeted therapy (YANG et al., 2019). A study showed that erianin inhibited the proliferation of MDA-MB-231 and EFM-192A cells in a dose-dependent manner, with *IC*_50_ of 70.96 and 78.58 nM, respectively. Erianin at 160 nM also increased the expression of Cyt-c, PARP, Bax, cleaved caspase-3, and -9, and decreased the expression of p-PI3K and p-Akt. The above effects could be reversed by PI3K agonist (SC79), further confirming that the anti-tumor effect of erianin is closely related to attenuating PI3K/Akt pathway (XU et al., 2021). Meanwhile, Su et al. reported that erianin significantly suppressed the proliferation of Huh7 cells, with *IC*_50_ of 37.3 nmol/L at 48 h. And erianin-induced Huh7 cell apoptosis and G_2_/M phase arrest. 78.5 nmol/L erianin markedly upregulated the cleaved PARP, while downregulated the expression of p-Akt and Mcl-1 (Su et al., 2011). Furthermore, Yang et al. confirmed that 10 and 20 μM of erianin inhibited the proliferation, migration and invasion of HCC cells in a dose- and time-dependent manner. Actually, erianin at 20 μM obviously promoted the apoptosis of HCC cell, and significantly suppressed the expression of p-Akt, p-ERK, p-p38 (YANG et al., 2020).

##### 2.1.1.5 JAK2/STAT3

JAK2/STAT3 pathway is a common signal pathway that transfers signals from extracellular to intracellular, and of course, is also a member of various biological processes in the cell (MENGIE AYELE et al., 2022). However, recently, it was reported that the abnormal activation of the JAK2/STAT3 pathway was closely associated with the growth, metastasis, angiogenesis, and other processes of many tumors, which has become a research hotspot in cancer treatment (KIU and NICHOLSON, 2012). Moreover, studies have shown that multidrug resistance (MDR) was the main cause of chemotherapy failure in many cancers, and the high expression of drug efflux protein P-gp was one of the main causes of MDR (CHOI and YU, 2014; AMAWI et al., 2019). Besides, the expression of P-gp was regulated by the STAT3 signal pathway (LIU et al., 2021). Su et al. found that the *IC*_50_ of erianin-resistant oxaliplatin-resistant cell line HCT116/LOHP was 10 times that of HCT116 cells, and erianin could enhance the sensitivity of drug-resistant strains to oxaliplatin. 200 μM erianin downregulated the expression of Bcl-2, CyclinD1, C-Myc, and p-STAT3 of JAK2/STAT3 signaling pathway-related protein, and also significantly decreased the expression of P-gp (SU et al., 2021).

##### 2.1.1.6 HIF-1α/PD-L1

Programmed death ligand 1 (PD-LI) is a key immune stimulator that synergistically represses the ability of T lymphocytes to induce apoptosis by binding to PD-1 on the surface of T lymphocytes (DONG et al., 2002). Virtually, PD-L1 is also a marker of HPV infection and is upregulated in cervical cancer cells (MEZACHE et al., 2015). All at once, and proinflammatory factor TNF-α activated HIF, further promoting tumor cell proliferation (NOMAN et al., 2014). It is reported that the proximal promoter of PD-L1 is directly bound to the hypoxia response element in HIF-1a (LI et al., 2017), in favor of speculating that HIF-1α/PD-L1 may be a new target for cancer immunity. Erianin could promote the degradation of lysosomes in Hela cells, further inhibiting the expression of PD-L1. Notably, 100 nM of Erianin inhibited the synthesis of HIF-1a via suppressing the expression of p-Akt, p-mTOR, p-4EBP1 and accelerating the expression of p-GSK3β, MTF, and TFE3. Simultaneously, erianin downregulated the expression of RAS/Raf/MEK/MAPK-ERK pathway-related proteins p-c-Raf, p-MEK, p-ERK, p-p38, and p-JUK. Besides, erianin also reduced the interaction between RAS and HIF-1a. Even erianin could significantly inhibit the proliferation, migration, invasion, and angiogenesis of Hela cells mediated by PD-L1. And the *in vivo* experiment further confirmed the anti-tumor effect of erianin (YANG et al., 2021).

##### 2.1.1.7 PPT1/mTOR

MTOR is a serine/threonine kinase that regulates cellular processes such as protein synthesis, metabolism, aging, regeneration, and autophagy (MURUGAN, 2019). Over the past few decades, many studies have shown that mTOR is activated in various tumors, thereby the use of mTOR signaling pathways and mTOR inhibitors has received widespread attention (HUA et al., 2019; DANESH PAZHOOH et al., 2021). Luo et al. reported that erianin could inhibit the survival rate, and G_2_/M arrest and promote apoptosis in OSCC cells (WSU-HN4, SCC-9, and CAL-27 cells). And erianin also significantly increased the formation of autophage, but decreased the function of autolysosome. Meanwhile, PPT1 was high-expressed in OSCC through an online database. And 100 nM of erianin significantly inhibited the expression of PPT1, p-mTOR, and p-4EBP1 *in vivo* and *in vitro*. Consequently, PPT1 played a key role in the inhibition of OSCC cell growth, indicating that erianin may have great potential in the treatment of OSCC (LUO et al., 2021).

##### 2.1.1.8 JNK/c-Jun

Multiple studies have shown that c-Jun amino-terminal kinase (JNK) signaling pathway plays a crucial role in cell proliferation, apoptosis, stress response, and the occurrence and development of many human diseases (ZHANG D. et al., 2021; WANG et al., 2021). JNK is activated and acts on downstream proteins, leading to inhibition of cell proliferation and apoptosis (KRAJARNG et al., 2015). Therefore, the activation of JNK pathway has become an important way for drugs to inhibit tumors. Erianin significantly inhibited the proliferation of MDA-MB-231 and MDA-MB-468 cells, and promoted cell shrinkage and nuclear fragmentation. Erianin upregulated the expression of PARP, caspase-3, caspase-8, and Bax. However, these effects could be partially reversed by JNK inhibitor SP600125, suggesting that the anti-breast cancer effect was related to the activation of the JNK/c-Jun signaling pathway (Zhao et al., 2020).

##### 2.1.1.9 p38 MAPK

MAPK signaling pathway participated in regulating a variety of cellular activities, including survival, differentiation, proliferation, and neuronal death (MARTíNEZ-LIMóN et al., 2020). A previous study showed that erianin had different degrees of inhibition on C918 and MUM-2B uveal melanoma cells. And erianin at 5, 10, and 20 μM promoted apoptosis and G_2_/M arrest in a concentration-dependent manner. Meanwhile, it significantly decreased the expression of p38 MAPK, and PARP, while evidently increasing the expression of p21, cleaved PARP, and cleaved caspase-3 with the increase in concentration (Guiping and Wang, 2019). Furthermore, Si et al. reported that 20, 40 and 80 nM of erianin induced apoptosis of Jurkat cells and upregulated the expression of the p38 signal pathway protein, including p-p38, γ H2AX, cleaved caspase-3, -9, and Bax (Xuyan et al., 2020). In general, the activation of p38 MAPK signal pathway is conducive to the anti-tumor effect of erianin in cancer treatment.

The Green: Represents the inhibition of erianin, and the Red: Represents the promotion of erianin.

#### 2.1.2 Cell cycle

Cell cycle is a highly organized and systematic process to ensure the integrity of genetic material during cell division. It involves the regulation of a sequence of cellular processes, including signals related to growth regulation, protein signals for monitoring genetic integrity, etc (DALTON, 2015). The cell cycle could be divided into two phases: interkinesis (G_1_, S, and G_2_ phases) and division phase M. And cyclin-dependent kinase (CDK) was activated at different stages of the cell cycle. For example, CDK1 was activated at G_2_ and M stages, CDK2 was activated at G_1_ and S stages, and CDK4 and CDK6 were activated at the G_1_ stages. In parallel, CDKS associated with cyclins form complexes that regulate the cell cycle (Liu et al., 2015). Erianin decreased osteosarcoma (OS) cell viability in a time- and dose-dependent manner. The *IC*_50_ values with erianin treatment for 24 h, 48 h, and 72 h were 58.19 nM, 40.97 nM and 26.77 nM in143B cells, while the *IC*_50_ values were 88.69 nM, 44.26 nM and 17.20 nM in MG63.2 cells, respectively. Synchronously, it significantly inhibited the proliferation of OS cells, and increased the number of the G_2_/M phase in 143B and MG63.2 cells. Furthermore, 25 and 50 nM of erianin upregulated cyclin B1, p-CDK1, p-Cdc25c, p21 and p27 (WANG et al., 2016). Interestingly, erianin also significantly induced G_2_/M arrest in PC3 cells and upregulated cyclin B1 and CDK1 (TRAPIKA et al., 2021). Taken together, erianin mainly inhibited the growth of tumor cells by inducing G_2_/M phase arrest. Therefore, it is a major strategy of anti-tumor therapy to induce cell cycle arrest and inhibit the unlimited proliferation of cancer cells in response to the cycle imbalance of cancer cells.

#### 2.1.3 Autophagy

Autophagy is an evolutionarily conserved vital process in the cell for the turnover of intracellular materials, which engulfs its cytoplasmic proteins or organelles and wraps them into vesicles. It fuses with lysosomes to form autophagic lysosomes, and degrades their contents, finally recycling them into the cytoplasm (LI et al., 2020). What’s more, autophagy is involved in mediating cancer development, preventing cancer cells from damage, promoting cancer metastasis, and inhibiting cancer therapy (FERRO et al., 2020). Remarkably, Chen et al. reported that 100 μM erianin could promote the formation of autophagosomes in SAS and SCC9 cells. Compared with the 0 μM group, erianin (25, 50, and 100 μM) increased the autophagy induction rate of SAS and SCC9 cells. Simultaneously, erianin dramatically upregulated the expression of LC3-I and LC3-II, and memorably downregulated the expression of p62, indicating that it induced autophagy in OSCC cells (CHEN YT. et al., 2020).

#### 2.1.4 Ferroptosis

Ferroptosis, a novel non-apoptotic programmed cell death process, is characterized by high iron levels and the accumulation of intracellular lipid reactive oxygen species (ROS) (XU et al., 2019). It is well-known that ferroptosis is closely connected with the metabolism of cysteine, polyunsaturated fatty acids (PUFAs), and iron (MOU et al., 2019). Exhilaratingly, it was strongly demonstrated that 50 and 100 nM of erianin effectively reduced the cell viability, and induced cell death and G_2_/M arrest of H460 and H1299 cells. Compared with the control group, 25 μM of erianin inhibited the migration of lung cancer cells. Moreover, erianin induced cell death through ferroptosis, accompanied by ROS accumulation, lipid peroxidation and GSH depletion. In fact, it also suppressed the expression of HO-1, GPX4, CHAC2, SLC40A1, SLC7A11, and glutaminase, and promoted the expression of Transferrin. Nevertheless, ferroptosis inhibitors Fer-1 and Lip-1 could reverse erianin-induced death. Subsequently, it was further proven that the inhibition of Ca^2+^/CaM could bring on the reduction of iron death. In summary, the anti-tumor effect of erianin may be in cooperation with calcium/calmodulin-dependent ferroptosis, which is conducive to being developed into a new drug for the effective treatment of lung cancer (CHEN P. et al., 2020).

#### 2.1.5 Angiogenesis

**FIGURE 3:**
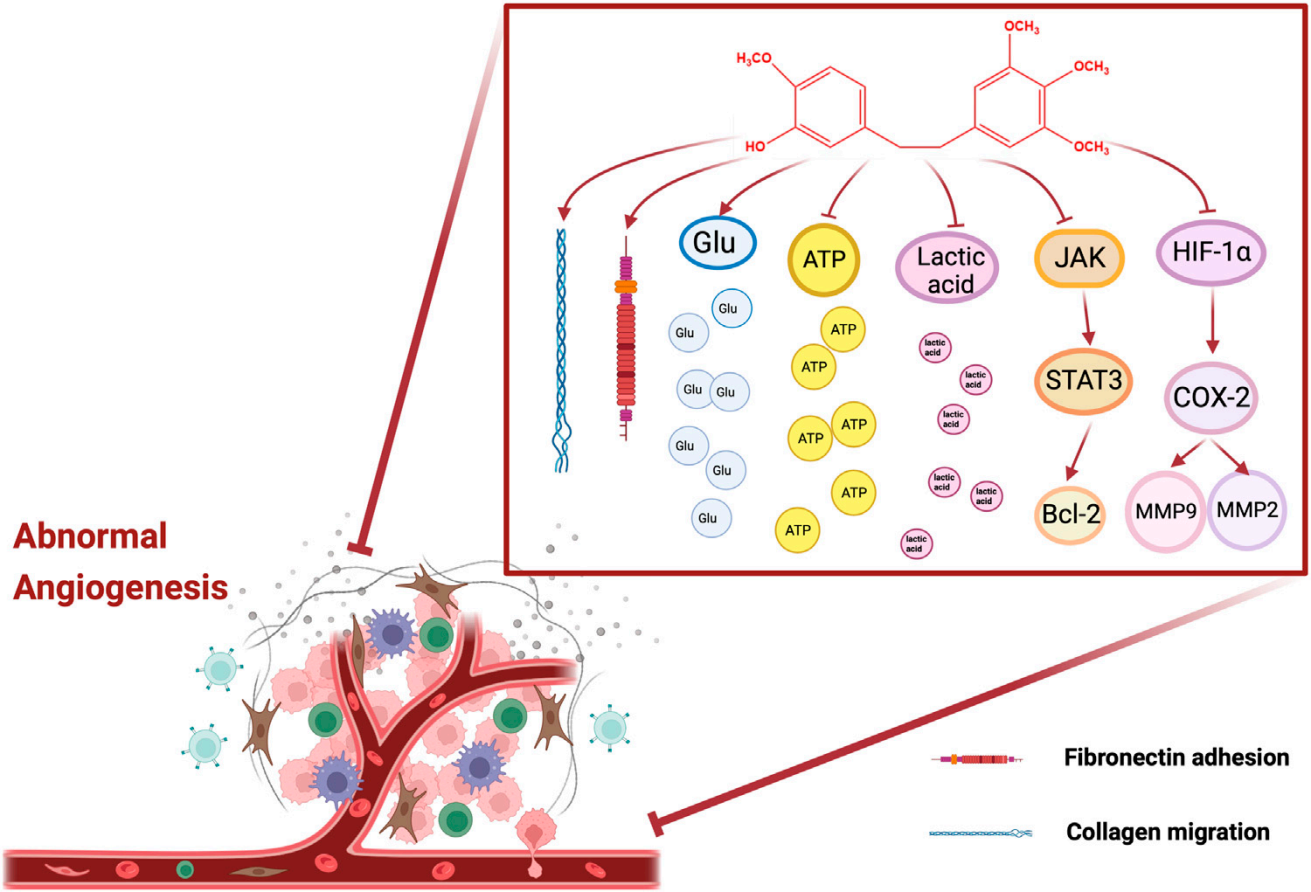
Molecular pathways involved in the angiogenesis of erianin.

Numerous studies demonstrated that anti-tumor angiogenesis was the pivotal target for cancer therapy (VIALLARD and LARRIVéE, 2017; LI Z et al., 2019). And it was discovered that tumor cells secreted a mass of pro-angiogenic factors, which contributed to the formation of an abnormal vascular network, thereby promoting tumor cell proliferation and survival. However, the application of antiangiogenic drugs was conducted to the inhibition of tumorigenesis. It mainly disrupted the vascular supply by blocking the VEGF/VEGFR signaling pathway, so that the tumor was deprived of the required nutrients and oxygen (RAMJIAWAN et al., 2017). Recent studies manifested that erianin has a certain therapeutic effect on anti-angiogenesis. Gong et al. found that 100 mg/kg of eranin could inhibit angiogenesis and tumor weight in tumor-bearing mice (xenografted with human hepatoma BEL7402 cells and melanoma A375 cells). And 10 μM of erianin abrogated basic fibroblast growth factor (bFGF)-induced angiogenesis in chicken embryos. In parallel, it inhibited the proliferation of HUVECs with an *EC*_50_ of (34.1 ± 12.7) nM. Moreover, erianin disrupted endothelial tube formation and abolished collagen migration and adhesion to fibronectin (GONG YQ. et al., 2004). Coincidentally, Gong et al. also reported that 10 nM of erianin decreased the acidification rate and survival rate of HUVECs, and restrained lactic acid production, glucose consumption and intracellular ATP content. Nonetheless, the above situation was significantly reversed by JNK/SAPK inhibitor SP600125, suggesting that erianin may inhibit endothelial metabolism via JNK/SAPK pathway (GONG Y. et al., 2004). Interestingly, the other study showed that erianin obviously reduced the activity and expression of indoleamine 2,3-dioxygenase (IDO), and simultaneously inhibited the IDO-induced invasions and migration ability of 2LL cells. Moreover, erianin blocked tube formation in HUVECs and the formation of VM in 2LL cells induced by IDO. Furthermore, erianin also downregulated the protein expression levels of p-STAT3, p-JAK2, MMP-2, MMP-9, COX-2, HIF-1α, and IL-6, indicating that erianin inhibited angiogenesis of lung cancer cells by targeting JAK2/STAT3 pathway and inhibiting IDO-induced angiogenesis (SU et al., 2017). On the whole, these studies suggested that the antitumor activity of erianin is associated with its ability to inhibit angiogenesis. The molecular targets of anti-angiogenesis of erianin mentioned in this article are shown in Figure 3.

### 2.2 Anti-inflammatory effect

Numerous studies have shown that the NF-κB family plays a crucial role in inflammation. And NF-κB cascades a variety of other signaling molecules and pathways, thereby synergistically exerting anti-inflammatory effects (HOESEL and SCHMID, 2013), referring toTLR4, STAT3, etc. Additionally, NLRP3 inflammasome is the research hotspot in recent years. The NLRP3 inflammasome is capable of recognizing pathogen-associated molecular patterns (PAMPs) or host-derived danger signaling molecules (DAMPs), recruiting and activating caspase-1, and further the activated caspase-1 cleaved pro-IL-1β and pro-IL-18, resulting in the increase of IL-1β and IL-18, eventually producing an inflammatory cascade (WANG et al., 2020).

#### 2.2.1 TLR4/NF-κB/STAT3

In 2020, a study had the highly emphatic sentence that erianin had an effective relieving effect on ulcerative colitis (UC). Erianin increased the weight and colon length of dextran sodium-sulfate-induced UC mice and reduced the activity index score. Besides, 20 mg/kg of erianin also decreased the content of ROS and ROS in the serum of UC mice, and also obviously descended the expression of TLR4, TRAF6, p-IKK α/β, p-IκB α, NF-κB p65, p-JAK2, p-Akt, and p-STAT3. Additionally, erianin reversed mucosal inflammation in colon tissue. To sum up, it showed that erianin’s anti-inflammatory activity was mediated by inhibiting TLR4/NF-κB/STAT3 signal transduction (DOU et al., 2020).

#### 2.2.2 NLRP3

A study in 2021 revealed that erianin inhibited the activation of NLRP3 inflammasome *in vitro* and *in vivo*, and blocked the assembly of NLRP3 inflammasome. The results indicated that erianin may target the ATP binding site of NLRP3, and then inhibited the oligomerization of NLRP3 and ATPase function. Simultaneously, in comparison with NLRP3 gene-deficient mice, erianin effectively inhibited the production of IL-1β and IL-18 and neutrophil influx induced by MSU in WT mice. Similarly, erianin also suppressed MSU-induced acute joint swelling and the production of IL-1β and IL-18 in the joint tissues of WT mice. Notwithstanding, erianin also increased the food intake, body weight, and blood sugar of WT mice. Ulteriorly, erianin has *in vitro* activity in synovial cells and monocytes from patients with AV infection and gout. Ultimately, these data supposed that erianin may be a potential new therapeutic compound against NLRP3-driven diseases (ZHANG X. et al., 2021).

### 2.3 Anti-diabetic retinopathy effect

Diabetic retinopathy (DR), the most common microvascular complication of diabetes, is one of the pathogenesis of visual impairment and blindness among working-age people worldwide. And visual impairment occurs in approximately 1/3 of patients with diabetes. Currently, the main effective methods used to treat DR include retinal laser photocoagulation, vitrectomy, corticosteroid or anti-VEGF therapy. Despite the efficacy of these approaches, the progression of the disease is in no condition to eliminate or reverse completely the retinal damage (WHITEHEAD et al., 2018; HE et al., 2019).

#### 2.3.1 ERK1/2–NF-κB

In 2019, erianin had an effective protective effect on the destruction of blood retinal barrier (BRB) in diabetic retinopathy. The results suggested that 10 mg/kg of erianin alleviated the damage of BRB in streptozotocin (STZ)-induced diabetic mice, and saved the reduction of claudin1 and cloddin expression in the retina. And it suppressed microglial activation and significantly reduced the expression of p-IκB, p-IKK, p-p65, and TNF-α and p-ERK1/2/t-ERK1/2 ratio *in vitro* and *in vivo*. In the bargain, erianin reduced cell glucose uptake. Molecular docking analysis showed the potential interaction between Erianin and glucose transporter 1 (GLUT1). Thus, the inhibition of erianin was reversed by the GLUT1 inhibitor (STF31). More importantly, erianin improved BRB damage in D-glucose stimulation of BV2 cells and TNF-α induced by stimulating APRE19 cells (ZHANG T. et al., 2019).

#### 2.3.2 ERK1/2-HIF-1α-VEGF/VEGFR2

At present, Yu et al. reported that erianin inhibited HG-induced RF/6A cell tube formation and migration, and reduced the expression of VEGF and HIF-1 α. Similarly, Erianin suppressed the protein expression of p-ERK1/2, p-VEGFR2, p-c-Raf, p-MEK1/2, p-p38, p-Akt, p-PI3K, p-mTOR, p-P70S6kinase. Additionally, erianin could eliminate retinal neovascularization, VEGF expression and microglia activation in STZ-induced hyperglycemia mice and oxygen-induced retinopathy (OIR) mice. In a word, the data suggested that blocking the ERK1/2-HIF-1α-VEGF/VEGFR2 signaling pathway might be an important mechanism of erianin in diabetic retinopathy therapy (YU et al., 2016).

### 2.4 Antibacterial effect

*Staphylococcus aureus* is a zoonotic pathogen that can cause a variety of diseases, including peritonitis, endocarditis, pneumonia, etc., which seriously threaten the life safety of humans and animals. Nevertheless, due to the abuse of antibiotics, drug-resistant bacteria such as methicillin-resistant *Staphylococcus aureus* (MRSA) have emerged, which is abundantly unfavorable for the clinical treatment of bacterial infections (Yuan et al., 2018; CHEUNG et al., 2021). Sorting enzyme A (SrtA), a kind of transpeptidase in gram-positive bacteria, is known as a potential antiviral drug target for the treatment of bacterial infections (FREUND and SCHWARZER, 2021). Actually, Ping et al. found that the *IC*_50_ of erianin against SrtA of *staphylococcus aureus* was 20.91 ± 2.31 μg/mL. Meanwhile, it was also showed that erianin is directly bound to SrtA residues to inhibit the effect of SrtA. When erianin was co-cultured with *staphylococcus aureus*, 32 and 64 μg/mL of erianin inhibited the activity of *staphylococcus aureus* binding to fibronectin and biofilm formation. Furthermore, erianin could improve the survival rate of mice infected with *staphylococcus aureus* through tail vein injection *in vivo* (OUYANG et al., 2018).

### 2.5 Antipsoriasis

Psoriasis is a chronic recurrent immune inflammatory skin disease, mainly including excessive proliferation and abnormal differentiation of keratinocytes (BOEHNCKE and SCHöN, 2015). Recent studies have shown that apoptosis of keratinocytes induced by increasing ROS level has promising potential in psoriasis treatment (KIM et al., 2017; ROUSSET and HALIOUA, 2018). Simultaneously, erianin (12.5, 25, and 50 nM) inhibited the proliferation and induced apoptosis of HaCaT cells. And erianin promoted the release of ROS, but was attenuated by a reactive oxygen species scavenger N-acetylcysteine (NAC). Besides, erianin at 50 nM apparently increased cleaved PARP, cleaved caspase-3, p-JNK, p-c-jun, and decreased p-Akt, p-mTOR, while these effects were also reversed with NAC. Ultimately, all the results indicated that the antipsoriasis effects of erianin may be mediated through the JNK/c-Jun/Akt/mTOR signaling pathway (MO et al., 2019).

## 3 Pharmacokinetics

Currently, pharmacokinetic studies of erianin have been conducted in rats and beagle dogs. The detailed pharmacokinetic parameters of these studies are shown in Table 2. Actually, erianin concentration in plasma decreased rapidly after intravenous administration in beagle dogs. And erianin had a relatively short half-life with a t_1/2_ of approximately 1.6 h. From the results of t_1/2_, CL/F, and V/F, it was confirmed that there was no obvious difference in the pharmacokinetic parameters of different concentrations of erianin (ZHOU et al., 2009). Additionally, the plasma concentration-time profiles of orally and intravenously administered rats were consistent with the non-compartmental model. Erianin was rapidly absorbed into plasma and reached a maximum concentration (148.5 ± 17.5) ng/mL at (0.6 ± 0.3) h after oral administration, and could also be quickly eliminated from the plasma. Nevertheless, its bioavailability was less than 50%, indicating that erianin was poorly absorbed in the gastrointestinal tract or had excessively high first-pass metabolism in life. Besides, the t_1/2_, CL, and MRT indicated that erianin could be rapidly eliminated from plasma after intravenous administration in rats. Notwithstanding, the half-life in both oral administration and intravascular administration was roughly similar, declaring that the half-life was not affected by the route of administration (YI and LAN, 2020).

**TABLE 2:** Pharmacokinetic studies of erianin

Species	Route of administration	Dosage (mg/kg)	t_1/2_ (h)	t_max_ (h)	C_max_ (ng/mL)	F (%)	AUC_0∼∞_ (μg/L h)	AUC_0-last_ (ng·h/mL)	AUC_0-inf_ (ng·h/mL)	MRT (h)	MRT_0-last_ (h)	MRT_0-inf_ (h)	CL/F (L/h)	V/F (mg·mL/μg)	CL (L/kg·h)	Vd (L/kg)	References
Beagle dogs	i.v	7.5	1.41 ± 0.31	\	\	\	1021.3 ± 373.7	\	\	0.81 ± 0.12	\	\	0.0081 ± 0.0025	0.0067 ± 0.0027	\	\	ZHOU et al. (2009)
		15	1.66 ± 0.19	\	\	\	2305.1 ± 597.0	\	\	1.01 ± 0.18	\	\	0.0069 ± 0.0016	0.0067 ± 0.0010	\	\	
		30	1.60 ± 0.28	\	\	\	3952.1 ± 378.2	\	\	1.00 ± 0.14	\	\	0.0077 ± 0.0008	0.0076 ± 0.0007	\	\	
Rats	Oral	10	1.4 ± 0.1	0.6 ± 0.3	148.5 ± 17.5	8.7	\	261.4 ± 44.9	266.7 ± 45.0	\	1.6 ± 0.2	1.7 ± 0.2	\	\	39.3 ± 7.5	76.1 ± 15.7	YI and LAN (2020)
	i.v	2	1.5 ± 0.4	\	\	\	\	605.9 ± 51.9	607.2 ± 51.7	\	0.7 ± 0.1	0.8 ± 0.1	\	\	3.3 ± 0.3	7.2 ± 2.6	

## 4 Derivatives

**FIGURE 4:**
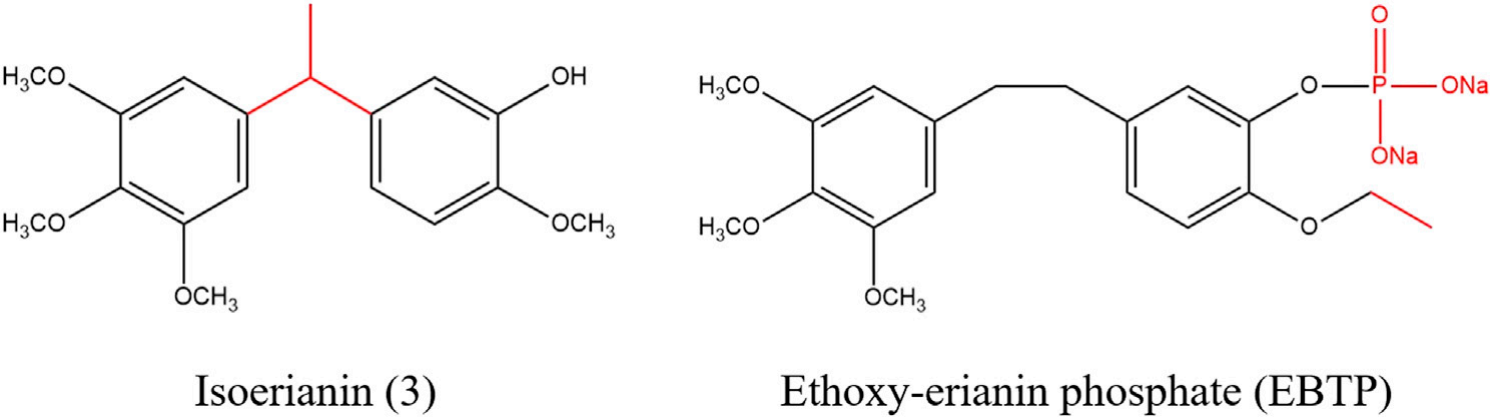
Chemical structure of erianin derivatives.

At present, a considerable number of studies have proved that erianin has multiple pharmacological effects, especially anti-tumor, which is potentially a natural product with good development prospects (ZHANG Y. et al., 2019). In order to solve the problem of low bioavailability of erianin, some derivatives related to erianin were synthesized by structural modification, some of which improved the bioavailability of drugs and enhanced pharmacological effects, as shown in Figure 4 (MESSAOUDI et al., 2011; LAM et al., 2012; YUAN et al., 2019). Messaoudi et al. obtained a series of isoflavone derivatives through structural modification. Among them, isoerianin (3) had a strong anti proliferation activity, which not only sensibly induced G_2_/M phase arrest and strongly promoted apoptosis in H1299 and K562 cells. Besides, isoerianin (3) could destroy the vessel-like structures formed by HUVECs *in vitro* (MESSAOUDI et al., 2011). Coincidentally, it was reported that erianin derivative ethoxy-erianin phosphate (EBTP) also exhibited extremely strong antitumor activity. 4μM of EBTP evidently induced G_2_/M phase arrest and inhibited migration of HUVECs cells. And EBTP also disrupted the vascular disrupting activity of the chorioallantoic membrane of fertilized chicken eggs. Simultaneously, EBTP inhibited the invasion and migration of IDO-2LL cells and inhibited tumor growth in lung cancer tumor-bearing mice. All in all, these data implied that EBTP may be a potent angiogenesis blocker and possess high bioavailability and good safety profile *in vivo* (YUAN et al., 2019).

## 5 Discussion

With the in-depth study of erianin, it has been clarified that erianin has greatly effective anti-tumor activity. The therapeutic effects of erianin are mainly achieved by promoting cell apoptosis, inducing cell cycle arrest, inhibiting angiogenesis, inducing cell autophagy and ferroptosis. Further researches showed that the interaction of multiple pathways participated in regulating the anti-tumor effect of erianin. The erianin molecular and cellular targets mentioned in this paper are shown in Table 1 and Figures 2, 3. Among them, the effect of erianin on promoting apoptosis were mainly associated with ERK1/2, Nrf2/NF-κB, PI3K/Akt, JAK2/STAT3, and other apoptosis pathways. Modulation of these pathways would induce the increase of the apoptosis-related factors Bax and caspase family, the decrease of Bcl-2, and phosphorylation of key proteins, such as JNK, Akt, ERK1/2, p38, mTOR, STAT3, and p65, while also further promoting apoptosis by causing mitochondrial damage. In parallel, oxidative stress and inflammation are also involved in regulating apoptosis. Inhibition of ROS excessive release, Nrf2 and HO-1 increase, as well as the activation of NF-κB and TNF-α was also beneficial for exerting the antitumor effects of erianin. Additionally, the inhibition effect of erianin on tumors was mainly mediated by inducing of G_2_/M phase arrest. It was advantageous for alleviating the imbalance of cell cycle to upregulate of cyclin B1, p-CDK1, p-Cdc25c, p21 and p27, and downregulate CDK1. Certainly, erianin could also accelerate the expression of LC3-I and LC3-II and induce the formation of autophage, thereby promoting cell autophagy. Actually, erianin significantly inhibited tumor angiogenesis. Erianin blocked the VEGF/VEGFR signal pathway to destroy the vascular supply. And It was effective for erianin to destroy the formation of endothelial tubes, and eliminate the migration of collagen and adhesion to fibronectin. Interestingly, ferroptosis also played a crucial role in the process of inducing cancer cell death. Erianin affected the metabolism of cysteine, PUFAs and iron. And erianin promoted ROS accumulation, lipid peroxidation, GSH depletion and transferrin expression, while also inhibited the expression of GPX4, CHAC2, SLC40A1, SLC7A11 and glutaminase. Simultaneously, it was inferred that the anti-inflammatory effect of erianin may be related to the regulation of TLR4/NF-κB/STAT3 and NLRP3 signaling pathways. Moverover, it was due to the inhibition of NF-κB and NLRP3 that erianin evidently reduced the phosphorylation of downstream pathway protein IKKα/β STAT3 and the expression of IL-1β And IL-18, further inhibiting the subsequent inflammatory cascade reaction. In addition, it would be of interest to the anti-diabetic retinopathy effect of erianin via ERK1/2–NF-κB and ERK1/2-HIF-1α-VEGF/VEGFR2 signaling pathway, which inhibited VEGF expression, retinal neovascularization and microglia activation. However, other pharmacological studies of erianin have not been found or the mechanism of action is not clear, and further research and development are urgently needed.

The pharmacokinetic parameters explicitly implied that erianin had the characteristics of low bioavailability, short half-life and poor water solubility (ZHOU et al., 2009; YI and LAN, 2020). In order to improve the above characteristics, it is advantageous to adopt a structural modification method or prepare a novel preparation (Zhang et al., 2019). Besides, research showed that the anti-tumor effect could be enhanced by modifying the 1, 2 and 5 positions of erianin (LAM et al., 2012; YUAN et al., 2019). Therefore, we speculate that selecting more appropriate substitutes to replace the groups at 1, 2 and 5 sites of erianin will contribute to research and develop new drugs with higher bioavailability, stronger water solubility, lower toxicity and side effects, and more effective and broader pharmacological effects.

On the whole, it is clear that erianin has potent antitumor effects, but studies on other pharmacological effects are lacking recently. Consequently, What’s urgent is that the mechanism of antitumor effect and other potential pharmacological effects require further in-depth study and development.
